# Changes in the Cardiac Index Induced by Unilateral Passive Leg Raising in Spontaneously Breathing Patients: A Novel Way to Assess Fluid Responsiveness

**DOI:** 10.3389/fmed.2022.862226

**Published:** 2022-04-11

**Authors:** Zhiyong Zhao, Zhongwei Zhang, Qionghua Lin, Lihua Shen, Pengmei Wang, Shan Zhang, Zhili Xia, Fangfang Li, Qian Xing, Biao Zhu

**Affiliations:** ^1^Department of Critical Care, Fudan University Shanghai Cancer Center, Shanghai, China; ^2^Department of Oncology, Shanghai Medical College, Fudan University, Shanghai, China

**Keywords:** fluid responsiveness, leg raising, cardiac index, pulse contour, volume expansion

## Abstract

**Background:**

Evaluation of fluid responsiveness in intensive care unit (ICU) patients is crucial. This study was to determine whether changes in the cardiac index (CI) induced by a unilateral passive leg raising (PLR) test in spontaneously breathing patients can estimate fluid responsiveness.

**Methods:**

This was a prospective study, and 40 patients with spontaneous breathing activity who were considered for volume expansion (VE) were included. CI data were obtained in a semirecumbent position, during unilateral PLR, bilateral PLR, and immediately after VE. If the CI increased more than 15% in response to the expansion in volume, patients were defined as responders.

**Results:**

The results showed that a unilateral PLR-triggered CI increment of ≥7.5% forecasted a fluid-triggered CI increment of ≥15% with 77.3% sensitivity and 83.3% specificity with and an area under the receiver operating characteristic (ROC) curve of 0.82 [*P* < 0.001]. Compared with that for bilateral PLR, the area under the ROC curve constructed for unilateral PLR-triggered changes in CI (Δ*CI*) was not significantly different (*p* = 0.1544).

**Conclusion:**

Δ*CI* >7.5% induced by unilateral PLR may be able to predict fluid responsiveness in spontaneously breathing patients and is not inferior to that induced by bilateral PLR.

**Trial Registration:**

Unilateral passive leg raising test to assess patient volume responsiveness: Single-Center Clinical Study, ChiCTR2100046762. Registered May 28, 2021.

## Background

Circulatory failure is very common in intensive care unit (ICU) patients. In individuals with circulatory failure, fluid resuscitation is one of the most basic interventions for treatment (1). Nevertheless, only 50% of severely ill patients with acute circulatory failure benefit from intravascular volume expansion (2, 3). The expansion of blood volume harbors harmful effects in the absence of preload dependence (4). Treatment involving excessive intravenous fluid might result in pulmonary and peripheral edema along with complications of the abdomen and other compartments and may impair oxygen diffusion (5–7). It is therefore of great importance to effectively evaluate the patient's volume capacity status in the clinic (8).

Several dynamic indices of fluid responsiveness based on heart-lung interaction-induced variations in left ventricular stroke volume can be used in mechanically ventilated patients but not in spontaneously breathing patients. Passive leg raising (PLR) is a simple way to estimate volume responsiveness with good accuracy and can be used in spontaneously breathing patients (8). However, there are possible limitations to the PLR test, of which a few have been demonstrated, such as significant atrophy of the patient's unilateral lower extremity, necessity of venous compression stockings, deep vein thrombosis of the lower extremities, and lower extremity amputation. In the situations above, a patient cannot perform a classic bilateral passive leg lift test but can perform a unilateral PLR test. A minifluid challenge (~100 ml of fluid) is able to predict stroke volume increases induced by 500 ml (9). It has been reported that bilateral PLR can recruit approximately 300 ml from the lower extremities (10), and therefore, we hypothesized that the blood volume mobilized by a unilateral PLR test may be sufficient to evaluate fluid responsiveness. No data are currently available concerning the unilateral PLR test in patients. This question is worth discussing.

In this study, we aimed to explore (1) whether cardiac index (CI) changes during a unilateral PLR could estimate fluid responsiveness in spontaneously breathing patients. (2) To compare changes in CI (Δ*CI*) triggered by classic bilateral PLR and unilateral PLR and the ability to estimate volume responsiveness.

## Patients and Methods

### Patients

This single-center, prospective clinical study (ChiCTR2100046762) was conducted from June 1^st^, 2021, to July 15^st^, 2021, at Fudan University Shanghai Cancer Center. The study was approved by the hospital's Ethics Committee (No. 2104233-4), and all enrolled patients provided written informed consent for the clinical trial and were willing to participate.

Forty patients with spontaneous breathing activity who were considered for volume expansion were included. The inclusion criteria were age over 18 years. The decision was made on the basis of clinical signs of inadequate tissue perfusion, such as (1) tachycardia; (2) mottled skin; (3) blood pressure <90/60 mmHg and/or mean arterial pressure of <75 mmHg; and (4) urine output below 0.5 ml/kg/minute for at least 2 h. When one of the inclusion criteria is met, we will judge whether the patient is in a state of hypovolemia, based on the patient's vital signs and clinical manifestations. Two experienced ICU doctors (at least 5 years of experience) are responsible for the patient enrollment. For disputes, all doctors will discuss together.

Patients were excluded if they had intra-abdominal hypertension (IAH, sustained elevation of intra-abdominal pressure above 12 mmHg), arrhythmia, pulmonary hypertension, severe heart valve disease, severe thoracic aortic abnormality, external cardiac pacemaker, head trauma, severe heart failure, or thrombotic stockings, or if they were uncooperative. Moreover, those not suitable for enrollment for other reasons, such as patients with clear hemorrhage or active bleeding and patients who needed immediate rescue, were also excluded.

### Study Design and Measurements

**Figure 1:**
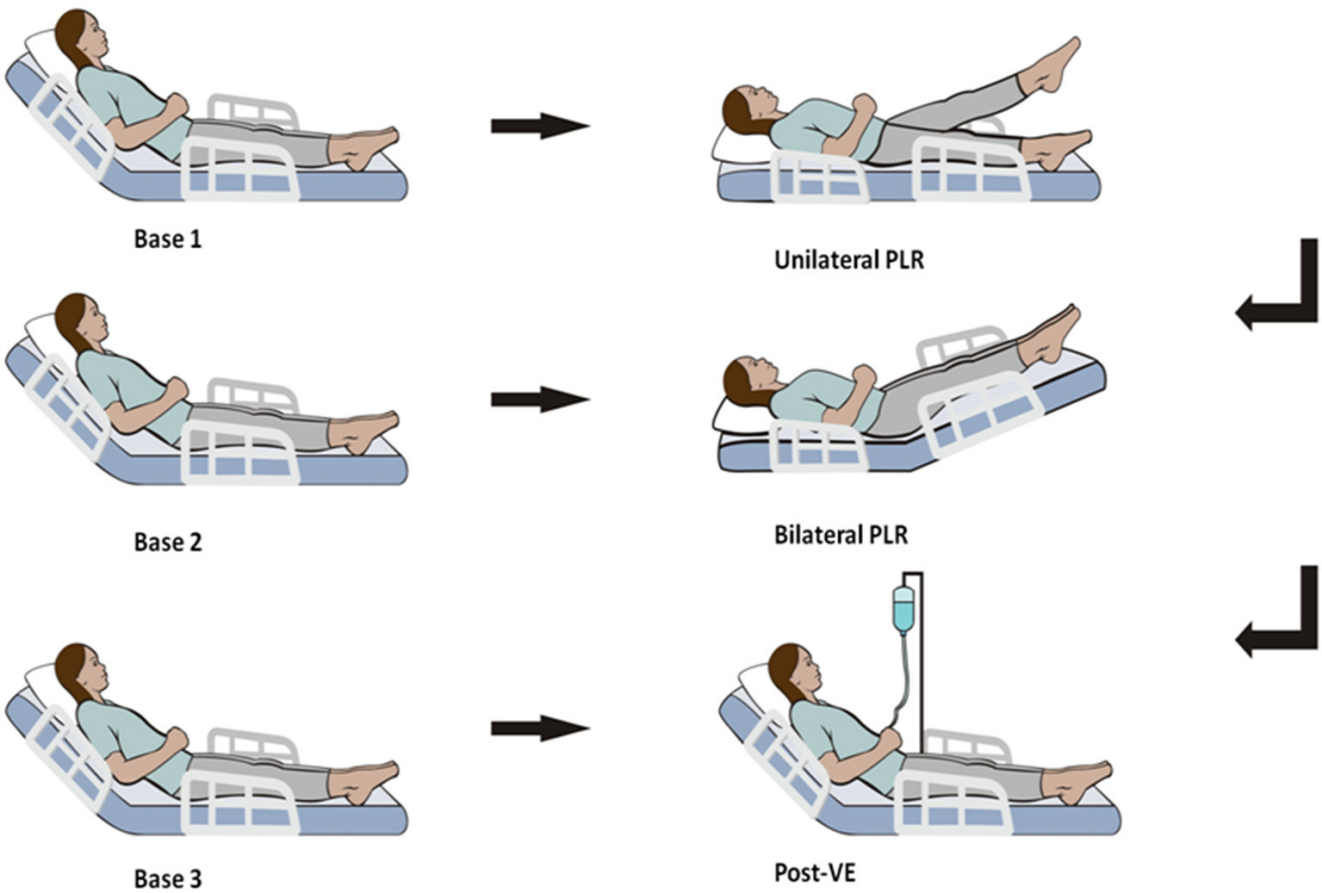
Study protocol. PLR, passive leg raising; VE, volume expansion.

Figure 1 illustrates the protocol steps of the current study. After 1 min of stabilization for each step, hemodynamic variables, such as heart rate, pulse pressure variation (PPV), systolic blood pressure, central venous pressure (CVP), CI, stroke volume variation (SVV), mean arterial pressure, stroke volume index (SVI) and diastolic blood pressure, were recorded. The initial value of the CI was estimated with a proprietary algorithm conducting an “autocalibration” by ProAQT/Pulsioflex, and data from the next steps were determined by pulse contour analysis with ProAQT/Pulsioflex.

Hemodynamic variables were measured during six sequential steps (Figure 1). The initial set of assessments was acquired in a semirecumbent position (45°) (named base 1), ensuring that the detected value was stable.

Next, we performed a unilateral PLR test. One of the legs was raised at a 45° angle by holding the patients' heels, while the patients' trunk and the other leg were in a supine posture. Therefore, the angle of the trunk with the lower raised leg remained unaltered at 135°. A second assessment set (named unilateral PLR) was documented at the maximal effect of unilateral PLR on the CI, occurring within 1 min.

The body position was then rendered to the base 1 posture, and the cardiac index was allowed to reach its baseline value. Then a third assessment set was documented (base 2).

To prevent possible pain from creating false-positives, the automatic technique was used for bed elevation. The patients' lower limbs were lifted to a 45° angle from the horizontal position, whereas the trunk was lowered to a horizontal position, and the angle of the trunk and the legs was still lifted at 135°. The fourth set of values (termed bilateral PLR) was measured when the CI reached its maximal value.

The patients were then shifted back to the base 1 position, and the fifth set of assessments was documented (base 3).

Finally, measurements were acquired immediately after volume expansion (VE) (500 mL of saline for 15–30 min) (designated post-VE).

Estimated CVP was measured at each study step, and the jugular venous pulse was evaluated to estimate CVP (11). Estimated CVP was measured at end-expiration and the averaged value from three sequential respiratory cycles was taken into account.

We haven't measure inferior vena cava. About half of the patients with upper abdominal surgery in our ICU, and the ultrasound quality were relatively poor. Moreover, the use of the inferior vena cava to assess volume responsiveness is controversial, and studies found that inferior vena cava showed poor accuracy to predict fluid responsiveness in spontaneous breathing patients (12–14).

### Hemodynamic Monitoring

The ProAQT/Pulsioflex (Pulsion Medical Systems, Munich, Germany, termed “Pulsioflex” hereafter) was used to estimate the CI from pulse contour analysis, without any external calibration. It was connected to a radial arterial catheter. The values of CI, SVI, SVV, and PPV were inferred from the device.

### Statistical Analysis

The calculation of the sample size was based on the comparison of two ROC curves (15). Expecting an AUC for the unilateral PLR-induced Δ*CI* of 0.70, anticipating an AUC for the bilateral PLR-induced Δ*CI* of 0.92, and selecting βas 0.2 andα as 0.05, we estimated that half of the patients would be preload responders. Thus, we planned to enroll 18 patients in each group.

After completing the study protocol, patients were divided into two groups: responders and non-responders to fluid loading. Patients with a CI increase of more than 15% by volume expansion from base 3 were classified as responders; otherwise, they were classified as non-responders.

The data distribution normality was screened with the Kolmogorov-Smirnov examination. Data are presented as the mean [standard deviation (SD)], median (interquartile range), or number (frequency in %).

The comparison of patient characteristics, medical history, and cause of circulatory failure between preload responders and non-responders was performed using a non-parametric Mann–Whitney U test for continuous variables and a chi-square test for categorical variables.

Comparison of hemodynamic variables between time points of the study was performed by the paired Student's *t* test or Wilcoxon test, based on the data distribution. Variables between preload responders and non-responders were analyzed using the two-sample Student's *t* test (normally distributed) or Mann-Whitney *U* test (non-normally distributed) as appropriate.

Receiver operating characteristic (ROC) curves were produced for unilateral PLR-induced changes in continuous variables (CI, PPV, SVI and SVV). The area under the ROC curve (AUC) for unilateral PLR-triggered Δ*CI* and bilateral PLR-triggered Δ*CI* were compared in all patients using a Hanley-McNeil test (15).

A two-tailed *p* < 0.05 showed statistical significance. Statistical analysis was implemented in MedCalc Statistical Software version 19.0.4 (MedCalc Software bvba, Ostend, Belgium; https://www.medcalc.org; 2019).

## Results

**Figure 2:**
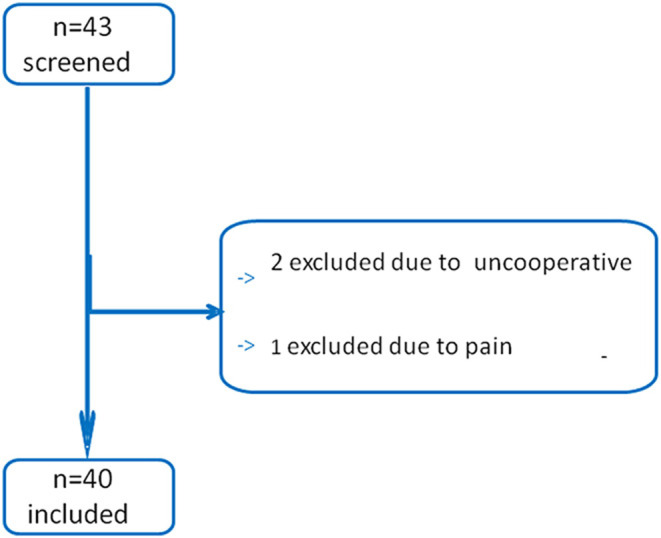
Flow of patients through the study.

Forty-three patients were screened in this study (Figure 2). Two patients were excluded due to being uncooperative with the test, and another patient was excluded because of pain when performing a unilateral PLR test. All the other patients were included. Finally, 40 patients were included and analyzed, as shown in Table 1.

**Table 1:** Characteristics of the study population.

**Parameters**	**Global population (40)**	**Non-responders (18)**	**Responders (22)**	***P*-value**
Patient's characteristics:				
Age (year)	59 ± 10	55 ± 8	61 ± 10	0.034
Male (*n*, %)	28 (70)	12 (30)	16 (40)	0.681
Weight (kg)	62.50 ± 9.19	61.28 ± 10.19	61.50 ± 8.53	1.000
Body mass index (kg/m^2^)	22.21 ± 2.81	21.93 ± 2.95	22.61 ± 2.71	0.568
Apache II (ICU admision)	7 ± 3	8 ± 4	7 ± 2	0.897
Death in the ICU (*n*, %)	4 (10)	3 (7.5)	1 (2.5)	0.209
Mechanical ventilation (*n*, %)	8 (20)	3 (7.5)	5 (12.5)	0.074
Medical history:				
Congestive heart failure (*n*, %)	7 (17.5)	3 (7.5)	4 (10)	0.015
Chronic respiratory insufficiency (*n*, %)	5 (12.5)	2 (5)	3 (7.5)	0.037
Abdominal surgery (*n*, %)	8 (40)	3 (7.5)	5 (12.5)	0.074
Thoracic surgery (*n*, %)	4 (10)	2 (5)	2 (5)	0.044
ARDS (*n*, %)	8 (20)	4 (10)	4 (10)	0.050
Hypertension (*n*, %)	4(10)	2(5)	2(5)	0.044
Diabetes (*n*, %)	2(5)	1(2.5)	1(2.5)	0.079
Chronic renal failure (*n*, %)	2(5)	1(2.5)	1(2.5)	0.079
Cause of circulatory failure:				0.49
Hypovolemic shock (*n*, %)	19 (47.5)	8 (20)	11 (27.5)	
Cardiogenic shock (*n*, %)	7 (17.5)	4 (10)	3 (7.5)	
Septic shock (*n*, %)	7 (17.5)	3 (7.5)	4 (10)	
Obstructive shock (*n*, %)	2 (5)	0 (0)	2 (5)	
Anaphylactic shock (*n*, %)	1 (2.5)	0 (0)	1 (2.5)	
Other causes (*n*, %)	4 (10)	3 (7.5)	1 (2.5)	

*ICU, intensive care unit; ARDS, acute respiratory distress syndrome*.

No patients received β-blockers. Every patient was breathed spontaneously. Eight patients (20%) were intubated and ventilated, and pressure support was in ventilation mode (fraction of inspired oxygen = 35 ± 5%, inspiratory pressure = 10 ± 4 cmH2O, and positive end-expiratory pressure = 5 cmH2O). Thirty-two patients were not intubated.

Twenty-two patients responded to the volume expansion, and 18 were non-responders. The impacts of unilateral PLR, bilateral PLR, and the expansion of volume on hemodynamic variables in responders and non-responders are shown in Table 2. As shown in Table 3, we found that unilateral PLR, the bilateral PLR test, and VE induced significant differences in Δ*CI* and Δ*SVI* between preload responders and preload non-responders.

**Table 2:** Evolution of hemodynamic parameters in preload responders and non-responders.

**Variable**	**Baseline1**	**Unilateral passive leg raising**	**Baseline 2**	**Bilateral passive leg raising**	**Baseline 3**	**Post volume expansion**
**Heart rate (beats/min)**						
Preload responders	105 ± 12	105 ± 11	106 ± 11	103 ± 11	106 ± 12	96 ± 11^*^
Preload non-responders	107 ± 14	108 ± 11	107 ± 14	107 ± 14	107 ± 15	106 ± 12
**Systolic arterial pressure (mmHg)**						
Preload responders	108 ± 12	108 ± 12	109 ± 14	115 ± 12^*^	108 ± 13	124 ± 12^*^
Preload non-responders	108 ± 14	109 ± 14	108 ± 13	110 ± 14	109 ± 12	110 ± 15
**Diastolic arterial pressure (mmHg)**						
Preload responders	58 ± 9	59 ± 8	58 ± 7	57 ± 9	57 ± 7	62 ± 8^*^
Preload non-responders	62 ± 8	62 ± 8	60 ± 6	61 ± 7	61 ± 6	60 ± 6
**CVP (mmHg)**						
Preload responders	5 (5–6)^*^	7 (6–8)^*^^!^	5 (4–6)^*^	7 (6–8)^*^^–^	6 (5–6)^*^	7 (6–9)^*^^#^
Preload non-responders	9 (8–10)^*^	10 (9–11) ^*^^!^	9 (8–9)^*^	10 (9–11)^*^^–^	9 (8–10)^*^	10 (8–11)^*^^#^
**SVV (%)**						
Preload responders	15 ± 7	15 ± 7	15 ± 6	14 ± 7–	16 ± 7	15 ± 7
Preload non-responders	12 ± 5	13 ± 4	13 ± 5	11 ± 4–	13 ± 5	12 ± 4
**SVI (ml/m** ^**2**^ **)**						
Preload responders	31 ± 9	34 ± 10	31 ± 9	46 ± 10	31 ± 7	47 ± 8#
Preload non-responders	31 ± 8	33 ± 9	32 ± 9	34 ± 9	32 ± 8	34 ± 8
**PPV (%)**						
Preload responders	20 ± 5	21 ± 5	19 ± 6	20 ± 7	23 ± 6	21 ± 5
Preload non-responders	11 ± 4	11 ± 4	12 ± 3	12 ± 4	14 ± 4	13 ± 4
**CI (L/min/m** ^**2**^ **)**						
Preload responders	3.25 ± 0.70	3.59 ± 0.71^!^	3.24 ± 0.63	3.79 ± 0.89–	3.20 ± 0.92	4.02 ± 1.03#
Preload non-responders	3.31 ± 0.66	3.51 ± 0.80^!^	3.36 ± 0.69	3.65 ± 0.69–	3.28 ± 0.83	3.69 ± 1.21#

* *p < 0.05 between preload responders and non-responders*.

! *p < 0.05 VS baseline 1*.

– *p < 0.05 VS baseline 2*.

# *p < 0.05 VS baseline 3*.

*CVP, Central Venous Pressure; SVV, Stroke Volume Variation; SVI, Stroke Volume Index; PPV, Pulse Pressure Variation; CI, Cardiac Index*.

**Table 3:** Indices of preload responsiveness in preload responders and non-responders.

**Variable**	**Effect of unilateral PLR**	**Effect of bilateral PLR**	**Effects of VE**
**ΔCI (% change)**			
Preload responders	10 ± 4	17 ± 5	20 ± 8
Preload non-responders	6 ± 2	9 ± 3	13 ± 7
P preload responders preload vs. non-responders	<0.001	<0.001	<0.001
**ΔSVV (% change)**			
Preload responders	−3 ± 18	5 ± 23	3 ± 26
Preload non-responders	10 ± 50	11 ± 18	5 ± 27
P preload responders vs. preload non-responders	0.819	0.476	0.757
**ΔSVI (% change)**			
Preload responders	11 ± 5	17 ± 6	19 ± 7
Preload non-responders	6 ± 2	9 ± 4	12 ± 8
P preload responders vs. preload non-responders	<0.001	<0.001	0.001
**ΔPPV (% change)**			
Preload responders	6 ± 26	8 ± 31	9 ± 31
Preload non-responders	−14 ± 43	7 ± 26	8 ± 26
P preload responders vs. preload non-responders	0.312	0.638	0.492

*PLR, passive leg raising; VE, volume expansion; ΔCI, percent changes in cardiac index; ΔPPV, percent changes in pulse pressure variation; ΔSVI, percent changes in stroke volume index; ΔSVV, percent changes in stroke volume variation*.

**Figure 3:**
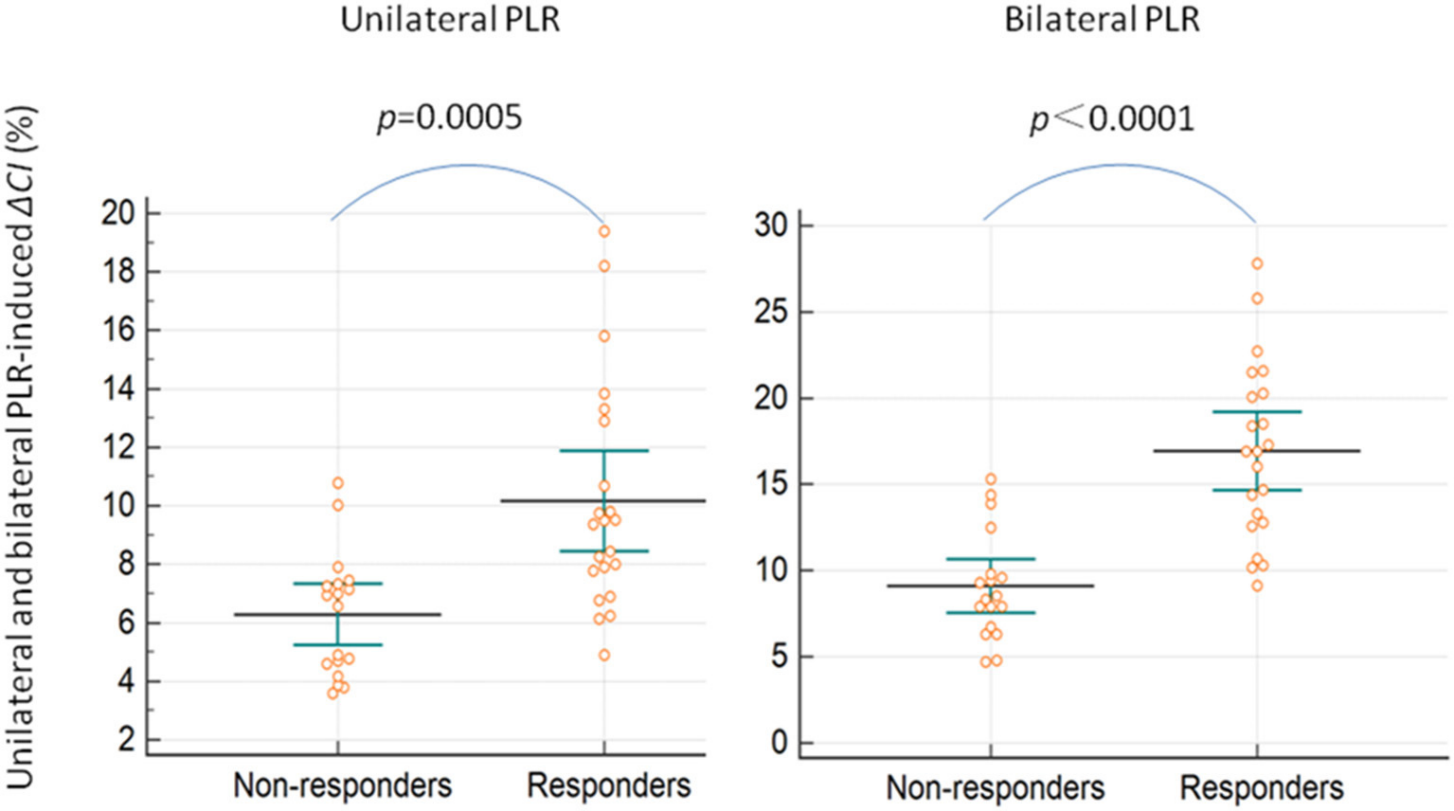
Unilateral and bilateral PLR-induced ΔCI in preload responders and non-responders. ΔCI, percent changes in the cardiac index; PLR, passive leg raising.

**Figure 4:**
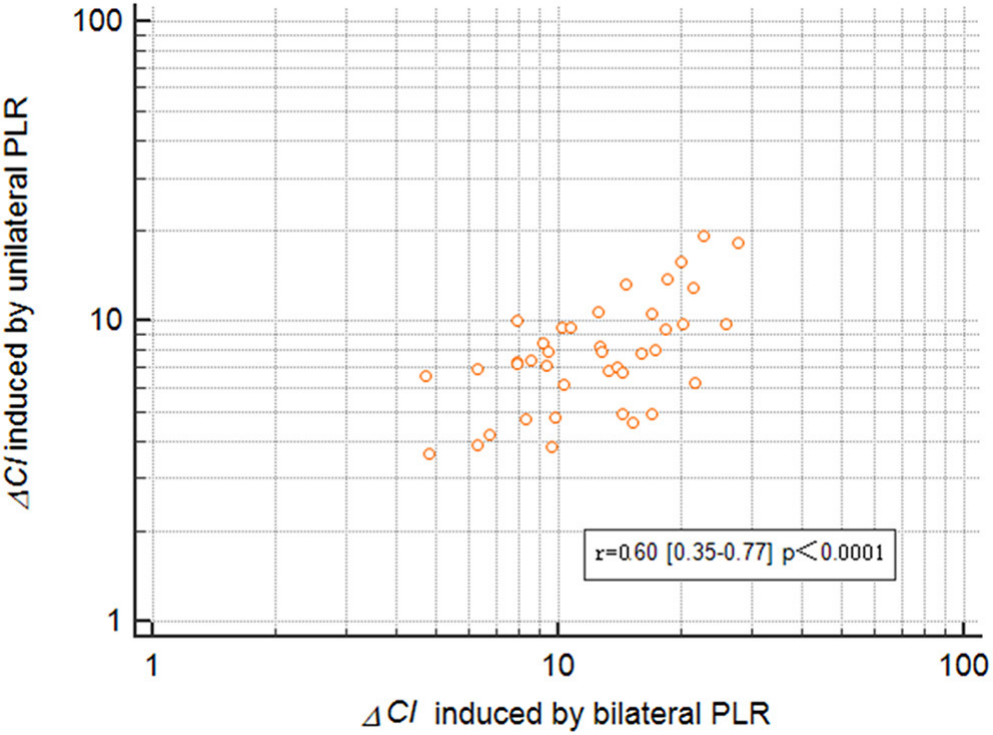
Correlation ΔCI between inducetion by unilateral and bilateral PLR. ΔCI, percent changes in the cardiac index; PLR, passive leg raising.

The maximal impact of PLR on the CI was detected within 1 min in all patients. Δ*CI* triggered by the unilateral leg raise test was significantly higher in responders than in non-responders (*p* = 0.0005; Figure 3). In responders, the CI increased by 10.2 (8.4–11.9) % from baseline to unilateral PLR. In non-responders, the CI increased by 6.3 (5.2–7.3) % from baseline to unilateral PLR. In all patients, Δ*CI* triggered by the bilateral leg raise test was significantly higher in responders than in non-responders (*p* < 0.0001; Figure 3). In responders, the CI increased by 16.9 (14.6–19.2) % from baseline to bilateral PLR. In non-responders, the CI increased by 9.1 (7.5–10.6) % from baseline to bilateral PLR. A correlation [r = 0.60 (0.35–0.77), *p* < 0.0001] between Δ*CI* induced by unilateral and bilateral PLR tests (Figure 4) was found.

**Figure 5:**
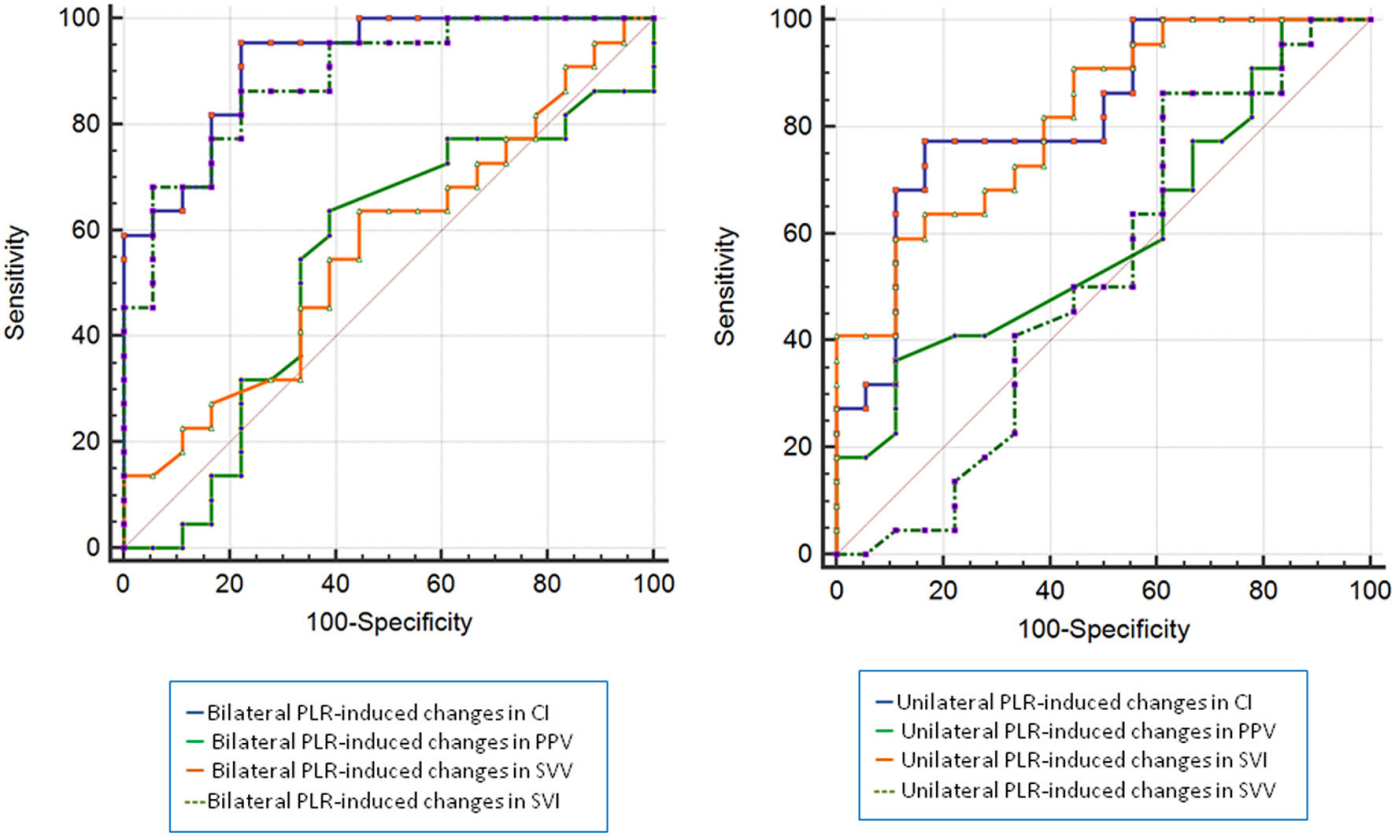
ROC curves comparing changes in CI, PPV, SVI, and SVV to discriminate responders and non-presponders (left: bilateral PLR, right: unilateral PLR). CI, cardiac index; PPV, pulse pressure variation; SVI, stroke volume index; SVV, stroke volume variation; ROC, receiver operating characteristic.

As shown in Figure 5 and Table 4, the AUC established for the unilateral and bilateral PLR-triggered changes in PPV and SVV was significantly lower than that established for the unilateral PLR-triggered Δ*CI* and Δ*SVI*.

**Table 4:** Diagnostic ability of the unilateral and bilateral PLR-induced changes in CI, PPV, SVI and SVV to detect preload responsiveness.

**Variable**	**AUC**	***p*-value verse 0.50**	**Best cutoff value**	**sensitivity**	**specificity**	**positive predictive value**	**Negative predictive value**	**Positive likehood ratio**	**Negative likehood ratio**	**Youden index**
Unilateral PLR-induced Δ*CI* (%)	0.82	<0.0001	7.5	77.2	83.3	85.0 (66.3–94.2)	75.0 (57.5–86.9)	4.64 (1.6–13.3)	0.27 (0.1–0.6)	0.6061
Unilateral PLR-induced Δ*PPV* (%)	0.60	0.2896								
Unilateral PLR-induced Δ*SVI* (%)	0.82	<0.0001	7.9	59.1	88.9	86.7 (62.7–96.2)	64.0 (51.2–75.1)	5.32 (1.4–20.6)	0.46 (0.3–0.8)	0.4798
Unilateral PLR-induced Δ*SVV* (%)	0.52	0.8256								
Bilateral PLR-induced Δ*CI* (%)	0.92	<0.0001	9.8	95.5	77.8	84.0 (68.8–92.6)	93.3 (67.0–99.0)	4.30 (1.8–10.2)	0.058 (0.008–0.4)	0.7323
Bilateral PLR-induced Δ*PPV* (%)	0.54	0.6441								
Bilateral PLR-induced Δ*SVI* (%)	0.89	<0.0001	11.0	86.4	77.8	82.6 (66.3–92.0)	82.4 (61.3–93.2)	3.89 (1.6–9.4)	0.18 (0.06–0.5)	0.6414
Bilateral PLR-induced Δ*SVV* (%)	0.57	0.4567								

*PLR, passive leg raising; AUC, area under the receiver operating characteristic curve; ΔCI, percent changes in cardiac index; ΔPPV, percent changes in pulse pressure variation; ΔSVI, percent changes in stroke volume index; ΔSVV, percent changes in stroke volume variation n = 40; mean (95% confidence interval), p-value to the AUC*.

**Figure 6:**
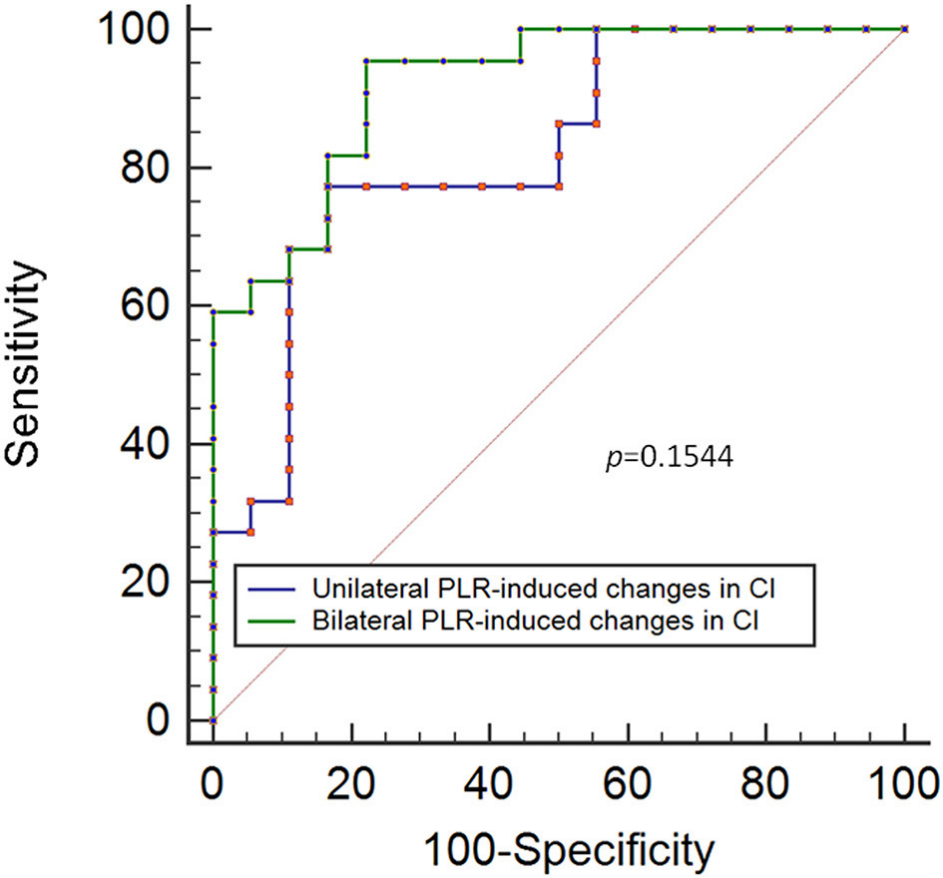
ROC curves showing the ability of changes in CI induced by unilateral PLR and bilateral PLR to predict an increase in the CI of ≥ 15% during volume expansion. ROC: receiver operating characteristic; CI, cardiac index; PLR, passive leg raising.

The results show that a unilateral PLR-triggered CI increment of ≥7.5% forecasted a fluid-triggered CI increment of ≥15% with 77.3% sensitivity and 83.3% specificity. Meanwhile, bilateral PLR-triggered increases in CI that were ≥9.8% forecasted a fluid-triggered CI increment of ≥15% with 95.5% sensitivity and 77.8% specificity (Table 4 and Figure 5). The AUCs constructed for unilateral and bilateral PLR-triggered alterations in the CI were not significantly different (*p* = 0.1544) (Figure 6).

## Discussion

This prospective study found that CI changes induced by a unilateral PLR greater than approximately about 7.5% predicted fluid responsiveness and were not inferior to bilateral PLR. To the best of our knowledge, this is the first clinical trial to address this problem.

The classical bilateral PLR test triggers a sudden increase in cardiac preload because of blood autotransfusion from the lower limbs and the vast splanchnic territory, resulting in a cardiac output increase in patients that is dependent on the preload. Under physiological conditions, the volume of blood in the capacity veins of the lower limbs and the vast splanchnic territory, returned during the classic bilateral PLR, is estimated at 300 ml (10). For the unilateral PLR test, the blood volume recruited should be <300 ml, similar to the “mini-fluid challenge,” which can lead to a significant cardiac output response. This trial is based on the assumptions that a small quantity of fluid can remarkably raise the cardiac preload and that this rise in preload is adequate to test the preload dependence of the two ventricles (16), which is confirmed by the results.

Acutely, during unilateral PLR, the trunk was lowered, and the splanchnic blood volume likely participated in the increase in preload, not just the blood volume of the raised leg. In this regard, in future research, we may be able to assess the effects of lowering only the trunk on the CI, if we performed the PLR in two steps [lowering the trunk and then elevating the leg(s)]. There is no relevant published report on this topic yet.

PPV is the most studied and used dynamic index in clinical practice and is a reliable indicator of preload responsiveness in patients with mechanical ventilation >8 ml/kg without spontaneous breathing. Taccheri et al. (17) found that Δ*PPV* can detect preload responsiveness during a bilateral PLR test in patients with mechanical ventilated at <8 ml/kg without spontaneous breathing. Hamazaoui et al. (18) found that in mechanically ventilated patients with spontaneous breathing, the Δ*PPV* induced by bilateral PLR could predict fluid responsiveness with moderate accuracy. However, in our study, the results showed that in patients with spontaneous breathing, Δ*PPV* was not a reliable marker of preload responsiveness during unilateral or bilateral PLR tests. Only 20% of patients received mechanical ventilation in our study, in contrast with two other studies which all patients received mechanical ventilation. Patients with spontaneous breathing without positive pressure ventilation may experience small changes in cardiac loading condition. In these patients, higher Δ*PPV* might be predictive of fluid responsiveness, but threshold have not defined (19). Further explorations are needed to determine whether Δ*PPV* induced by PLR can assess preload responsiveness in patients with spontaneous breathing activity.

The unilateral PLR test has some significant advantages. Some special situations are encountered in a clinical setting, such as disorders affecting one of the lower limbs rendering patients unable to perform a bilateral passive leg lift test. At this time, a unilateral PLR test can be used to evaluate the patient's volume capacity. Furthermore, unlike a fluid challenge test that may induce fluid overload, unilateral PLR increases preload by transferring blood pooled in the lower extremities to the compartment. The fluid is reversible when the patient is returns to the semirecumbent position, similar to the bilateral PLR test. Unilateral PLR may avoid this issue and still provide good volume forecasting.

There are some limitations to this study. First, performing the PLR test requires the ProAQT/Pulsioflex to estimate CI, which is invasive. Second, when the unilateral PLR test was performed, one of the lower limbs was manually lifted by holding the patients' heels. However, the maneuver was performed gently to prevent possible pain from lifting the leg. One patient was still excluded because of pain. There were only seven patients with septic shock, and none were placed under vasopressor support. Thus, these findings cannot be extrapolated to patients with septic shock who receive vasopressor support. Finally, this study was conducted using pro-AQT algorithms, and therefore, our results cannot be extrapolated to other algorithms. The hemodynamic parameters were average values obtained during the last 12 s. At any timepoint, the values resulted from both the former autocalibration and the pulse contour assessment that was run afterward. Data lag was inevitable.

## Conclusion

Δ*CI* >7.5% induced by unilateral PLR may be able to predict fluid responsiveness in spontaneously breathing patients. In addition, the significance of this study may not lie in how accurately CI changes resulting from unilateral PLR can determine whether the volume response is positive, but may stem from the presentation of a new method that can be used to predict fluid responsiveness. This is especially true for patients who cannot undergo bilateral PLR, but are eligible for unilateral PLR.

## Data Availability Statement

The original contributions presented in the study are included in the article/supplementary material, further inquiries can be directed to the corresponding author.

## Ethics Statement

The studies involving human participants were reviewed and approved by the Ethics Committees of Shanghai Cancer Center, Fudan University, China. The patients/participants provided their written informed consent to participate in this study.

## Author Contributions

ZhiZ, ZhoZ, QL, and BZ: conception and design. LS, PW, and SZ: administrative support. ZX, QX, and FL: provision of study materials or patients. ZhiZ and ZhoZ: collection and assembly of data, data analysis, and interpretation. All authors wrote the manuscript and approved the final manuscript.

## Conflict of Interest

The authors declare that the research was conducted in the absence of any commercial or financial relationships that could be construed as a potential conflict of interest.

## Publisher's Note

All claims expressed in this article are solely those of the authors and do not necessarily represent those of their affiliated organizations, or those of the publisher, the editors and the reviewers. Any product that may be evaluated in this article, or claim that may be made by its manufacturer, is not guaranteed or endorsed by the publisher.

## Abbreviations

ICU: intensive care unit
ARDS: acute respiratory distress syndrome
SV: stroke volume
PLR: passive leg raising
CI: cardiac index
IAP: intra-abdominal pressure
PPV: pulse pressure variation
CVP: central venous pressure
SVV: stroke volume variation
SVI: stroke volume index
VE: volume expansion
SD: standard deviation
ROC: receiver operating characteristic
AUC: area under the ROC curve;
Δ*CI*: percent changes in cardiac index
Δ*PPV*: percent changes in pulse pressure variation
Δ*SVI*: percent changes in stroke volume index
Δ*SVV*: percent changes in stroke volume variation.
